# Transcriptomic profiling identifies NLRP3-associated inflammatory signatures in acute radiation enteritis and characterizes the protective effects of Qing Hua Yi Chang formula

**DOI:** 10.3389/fimmu.2026.1890536

**Published:** 2026-09-16

**Authors:** Yuanyuan Qin, Jing Li, Yifan Qiu, Jianming Chang, Li Li, Mianhua Wu, Cui Mao

**Affiliations:** 1 First Clinical Medical College, Nanjing University of Chinese Medicine, Nanjing, Jiangsu, China; 2 Department of General Surgery, Affiliated Nantong Hospital 3 of Nantong University, Nantong, China; 3 Research Center of Clinical Medicine, Affiliated Hospital of Nantong University, Nantong, China; 4 Department of Urology, Jiangsu Province Geriatric Hospital, Nanjing, Jiangsu, China; 5 School of Computer Science and Engineering, Southeast University, Nanjing, China; 6 Jiangsu Collaborative Innovation Center of Traditional Chinese Medicine Prevention and Treatment of Tumor, Nanjing University of Chinese Medicine, Nanjing, Jiangsu, China

**Keywords:** acute radiation enteritis, intestinal barrier, NLRP3 inflammasome, Qing Hua Yi Chang formula, transcriptomics

## Abstract

**Background:**

Acute radiation enteritis (ARE) is a common complication of abdominal or pelvic radiotherapy characterized by inflammatory activation and epithelial barrier dysfunction. However, the molecular mechanisms underlying ARE remain incompletely understood. This study investigated transcriptomic alterations associated with ARE and evaluated the protective effects of Qing Hua Yi Chang Formula (QHYCF).

**Methods:**

A murine model of ARE was established using abdominal irradiation followed by QHYCF treatment. Histopathological analysis, TUNEL staining, RT-qPCR, western blotting, and ELISA were performed to assess intestinal injury and inflammatory responses. Transcriptomic profiling combined with bioinformatic analyses was used to identify inflammatory signatures and pathways associated with ARE.

**Results:**

QHYCF attenuated radiation-induced intestinal injury, particularly at the medium dose. Transcriptomic analysis revealed dysregulation of immune- and inflammation-related pathways after irradiation and identified NLRP3-associated inflammatory signaling as a prominent inflammatory alteration in ARE. QHYCF treatment was associated with reduced NLRP3 expression and decreased serum levels of IFN-γ, IL-1β, and IL-18. In addition, QHYCF partially restored Claudin-1 expression, suggesting improvement in epithelial barrier integrity.

**Conclusion:**

ARE progression is closely associated with inflammatory activation and epithelial barrier disruption. NLRP3-associated inflammatory alterations and Claudin-1 changes may represent molecular features associated with radiation-induced intestinal injury. QHYCF was associated with attenuation of inflammatory responses and preservation of epithelial barrier integrity.

## 1 Introduction

Radiotherapy is a key component of treatment for many malignant tumors, and approximately 50% of cancer patients receive radiotherapy during the course of their disease (1). Due to the active proliferation of intestinal epithelial cells and their high sensitivity to ionizing radiation, radiation enteritis (RE) is recognized as a frequent and clinically significant adverse effect associated with abdominal and pelvic radiotherapy (2). More than 75% of patients undergoing pelvic radiotherapy experience acute intestinal reactions of varying severity during treatment. In some cases, the condition may further progress to chronic intestinal stenosis, fibrosis, or even perforation (3–5), seriously affecting patients’ quality of life and tolerance to radiotherapy. Current clinical management mainly relies on symptomatic and supportive therapies (6). Therefore, developing effective therapeutic interventions and elucidating the mechanisms driving ARE pathogenesis remain critical research priorities.

Traditional Chinese medicine (TCM) has been increasingly investigated as an adjunctive approach for the prevention and treatment of RE because of its multi-component and multi-target characteristics (7). Qing Hua Yi Chang Formula (QHYCF) consists of eight herbs, including *Astragalus membranaceus*, *Atractylodes macrocephala Koidz.*, *Sanguisorba officinalis*, *Dendrobium nobile*, *Sargentodoxa cuneata*, *Coix lacryma-jobi L.* var. *mayuen*, *Aucklandia lappa Decne.*, and *Glycyrrhiza uralensis* (Pharmacopoeia of the People’s Republic of China, 2020) (8). Several components of QHYCF have been reported to exhibit anti-inflammatory properties. For example, *Astragalus membranaceus* reduces pro-inflammatory cytokine release (9), *Atractylodes macrocephala* shows anti-inflammatory and antioxidative activities (10), and *Glycyrrhiza uralensis* regulates inflammatory responses through NF-κB and MAPK signaling pathways (11). These findings suggest that QHYCF may have therapeutic potential in alleviating radiation-induced intestinal injury. As a patented formulation developed by the Affiliated Hospital of Nanjing University of Chinese Medicine, QHYCF has been clinically applied in the prevention and management of RE (Patent No. ZL202410165838.X). Preliminary clinical observations indicate that QHYCF may alleviate ARE symptoms and improve patients’ quality of life. However, its underlying molecular mechanisms remain incompletely understood.

Recent advances in transcriptomic technologies provide new opportunities to investigate gene expression alterations and identify key molecular pathways involved in RE. In the present study, we established a murine model of ARE to evaluate the protective effects of QHYCF. Transcriptomic profiling combined with bioinformatic analysis was performed to identify molecular alterations associated with ARE, followed by experimental validation of inflammatory and epithelial barrier-related changes after QHYCF treatment.

## 2 Materials and methods

### 2.1 Drug preparation

QHYCF was prepared based on a traditional empirical prescription established at the Affiliated Hospital of Nanjing University of Chinese Medicine. The constituent herbal materials were obtained from the hospital and met the quality requirements for clinical use. The detailed composition of the formula is presented in **Table 1**. All herbal materials were mixed according to the specified proportions and immersed in distilled water (10-fold volume) for 1 h. The mixture was first heated to boiling and then decocted gently for 30 min. After filtration, the remaining residues were subjected to a second extraction using eight volumes of water following the same procedure. The combined filtrates were subsequently concentrated under reduced pressure with a rotary evaporator, yielding a final extract with a concentration of 4 g·mL^–1^.

**Table 1:** The components of QHYCF.

Chinese name	English name	Scientific name	Amount (g)	Plant part
Huang Qi	Astragalus root	*Astragalus membranaceus*	15	Root
Bai Zhu	Atractylodes rhizome	*Atractylodes macrocephala* Koidz.	12	Root
Di Yu	Great burnet root	*Sanguisorba officinalis*	12	Root
Shi Hu	Dendrobium stem	*Dendrobium nobile*	12	Stem
Da Xue Teng	Sargentodoxa vine	*Sargentodoxa cuneata*	12	Vine stem
Yi Yi Ren	Coix seed	*Coix lacryma-jobi* L. var. mayuen	12	Seed kernel
Mu Xiang	Costus root	*Aucklandia lappa* Decne.	6	Root
Gan Cao	Licorice root	*Glycyrrhiza uralensis*	6	Root & stem

### 2.2 Identification of active ingredients in QHYCF

The chemical constituents of QHYCF were characterized using ultra-performance liquid chromatography coupled with tandem mass spectrometry (UPLC-MS/MS). Detailed experimental procedures are described in **Supplementary Material 1**. Raw spectral data were analyzed with Compound Discoverer software (version 3.3, Thermo Fisher Scientific). Potential compounds were identified by comparing the obtained spectra with entries in the mzCloud database, and only those with a best-match score exceeding 60 were retained as candidate active components.

### 2.3 Experimental animals

Considering that estrous cycle variations and sex hormone fluctuations can influence inflammatory responses (12), only male C57BL/6J mice (6–8 weeks of age, weighing 18–20 g) were included in this study. The animals were purchased from Hangzhou Medical College (Production License No. SCXK (Zhe) 2019-0002) and maintained in the specific pathogen-free barrier facility of Nanjing University of Chinese Medicine (Use License No. SYXK (Su) 2018-0049). Environmental conditions in the animal facility were controlled at 24 ± 2 °C with 55 ± 5% relative humidity, under a 12 h light/12 h dark photoperiod. Throughout the study, mice were allowed unrestricted access to standard laboratory chow and sterilized drinking water. All experimental procedures involving animals complied with the ARRIVE guidelines, and the study protocol was reviewed and approved by the Animal Ethics Committee of Nanjing University of Chinese Medicine (Approval Nos. 202206A030, 202210A008).

### 2.4 ARE model construction

Mice were anesthetized via intraperitoneal injection of 1.25% tribromoethanol (0.2 mL/10 g body weight; AiBei, China, M2910). Once an appropriate anesthetic depth was confirmed, the animals were placed in a supine position on the irradiation platform. Abdominal irradiation was subsequently delivered using a VitalBeam medical linear accelerator. The mice were exposed to 6 MV X-rays, with the irradiation area beginning approximately 0.5–1 cm inferior to the xiphoid process. No lead shielding was used. Instead, abdominal irradiation was achieved through accurate animal positioning and radiation field definition using a clinical linear accelerator. The irradiation field was primarily restricted to the abdominal region. A single dose of 15 Gy was administered at a dose rate of 400 cGy/min, with a source-to-skin distance of 100 cm.

### 2.5 Animal grouping and treatment

Two independent batches of animals were used in the study. The first batch served for model establishment and validation, whereas the second batch was designed to evaluate therapeutic efficacy. In the first experiment, 30 mice were stratified according to body weight (18–20 g, interval of 0.66 g) using a stratified randomization procedure to ensure comparable weight distribution between groups. The animals were subsequently assigned randomly into two groups (*n* = 15 per group): a normal control group (NC) and an irradiation model group (IR). For the second experiment, 90 mice were stratified using the same weight-based method and randomly divided into six groups (*n* = 15 per group): NC, IR, a positive treatment group receiving compound glutamine (IRAC), and three QHYCF intervention groups including low-dose (IR-L), medium-dose (IR-M), and high-dose (IR-H). Animals were housed in cages containing five mice each and allowed to acclimate for one week before the start of the experimental procedures.

No active treatment was administered to animals in the NC group. Compound glutamine was selected as the positive control based on previous studies evaluating its use in radiation enteritis (13). The clinical dosage of QHYCF was derived from a patented formulation that specifies the recommended daily dose (Patent No. ZL202410165838.X). Mouse-equivalent doses were determined using the standard body surface area conversion method commonly applied in preclinical pharmacological research (14). Since the optimal dosage has not yet been confirmed through clinical trials, three dosing levels were established in this study to perform exploratory evaluation. Mice in the IRAC group received compound glutamine suspension (24.96 mg/mL) via oral gavage. QHYCF was administered at three concentrations: low dose (0.5655 g/mL), medium dose (1.131 g/mL), and high dose (2.262 g/mL). Both glutamine and QHYCF preparations were dissolved in distilled water and delivered by gavage at 0.2 mL per 10 g body weight. To maintain procedural consistency, mice in the NC group were given the same volume of distilled water. Animals were fasted for 8 h prior to irradiation. Drug administration was conducted in two stages: a 7-day pretreatment period before irradiation followed by 14 days of treatment after irradiation, with gavage performed twice daily. All groups except NC were subjected to abdominal irradiation. Blood samples and intestinal tissues were collected at 3.5, 7, and 14 days after irradiation, with five animals from each group euthanized by cervical dislocation following anesthesia at each time point for subsequent analyses. Survival was not evaluated as an outcome because animals were euthanized at predetermined sampling time points according to the experimental design.

### 2.6 Pathological analysis and TUNEL staining of mouse intestinal tissue

A 2-cm segment of ileal tissue located near the ileocecal junction was collected and fixed in 4% paraformaldehyde for 48 h. After fixation, samples were processed for paraffin embedding and sectioned into 4 μm-thick slices. Histopathological assessment was performed using hematoxylin and eosin (H&E) staining (Servicebio, China, G1076). The stained sections were mounted with neutral gum and examined under a light microscope for morphological evaluation. To further evaluate radiation-induced epithelial apoptosis, TUNEL staining was carried out on paraffin sections of the ileum using a TUNEL *In Situ* Apoptosis Detection Kit (Elabscience, E-CK-A320) following the manufacturer’s protocol. Day 7 was selected as a representative time point because the irradiated intestine exhibited evident pathological injury at this stage. Fluorescent images were obtained using a fluorescence microscope with identical exposure parameters applied to all experimental groups. For each group, intestinal sections from three randomly selected mice were analyzed. The apoptotic index was calculated as the proportion of TUNEL-positive nuclei relative to the total number of DAPI-stained nuclei, expressed as TUNEL^+^/DAPI^+^ × 100%.

### 2.7 Transcriptome sequencing and analysis

Total RNA was isolated from mouse intestinal tissues using TRIzol reagent according to the manufacturer’s protocol. RNA concentration and purity were measured with a NanoDrop spectrophotometer, and RNA integrity was assessed using an Agilent 4200 TapeStation system. Only samples that satisfied the required quality control criteria were used for subsequent library construction. Strand-specific sequencing libraries were prepared and sequenced on the Illumina NovaSeq™ 6000 platform using the paired-end 150 bp (PE150) mode. Library preparation and sequencing were carried out by Hangzhou Lianchuan Biotechnology Co., Ltd.

For transcriptomic analysis, intestinal tissues collected 7 days after irradiation from the NC and IR groups were subjected to bulk RNA sequencing (*n* = 3 per group). Raw sequencing reads were first subjected to quality control filtering, followed by alignment to the mouse reference genome, after which gene-level read counts were obtained. Differential gene expression between the IR and NC groups was analyzed using the DESeq2 package. Genes were considered differentially expressed when they met the criteria of FDR < 0.05 and |log_2_ fold change| > 2. Functional annotation of DEGs was performed through Gene Ontology (GO) and Kyoto Encyclopedia of Genes and Genomes (KEGG) enrichment analyses. In addition, Gene Set Enrichment Analysis (GSEA) was conducted based on the ranked gene list derived from the IR versus NC comparison. Visualization of the results included volcano plots, enrichment bar charts, and GSEA enrichment curves.

### 2.8 Detection of NLRP3 mRNA expression in intestinal tissues by RT-qPCR

Total RNA was isolated from intestinal tissues using TRIzol reagent (Invitrogen, USA) according to the manufacturer’s instructions. The extracted RNA was subsequently reverse-transcribed into complementary DNA (cDNA) using a reverse transcription kit (Thermo Fisher Scientific, K1622). Quantitative real-time PCR was performed using SYBR Green Master Mix (Thermo Fisher Scientific, A25743) following the recommended protocol. The sequences of all primers used in this study are listed in **Supplementary Table 1**.

### 2.9 Detection of NLRP3 and Claudin-1 protein expression in intestinal tissues by western blot

Total protein was extracted from mouse intestinal tissues, and protein concentrations were determined using a BCA protein assay kit. Equal amounts of denatured protein samples (40 μg per lane) were separated by 10% SDS–PAGE and subsequently transferred onto 0.22 μm PVDF membranes. After blocking, the membranes were incubated overnight at 4 °C with primary antibodies against NLRP3 (1:1000), Claudin-1 (1:2000), and β-actin (1:2000) (Abcam, Cambridge, UK). Following washing with 1× TBST, the membranes were incubated with a rabbit secondary antibody (1:10, 000) for 1 h at room temperature. Protein bands were detected using a ChemiDoc XPS imaging system and quantified with ImageJ software (version 1.51). The expression levels of target proteins were normalized to β-actin.

### 2.10 ELISA determination of IFN-γ, IL-18, and IL-1β levels in mouse serum

Serum concentrations of IFN-γ, IL-18, and IL-1β were determined using commercially available ELISA kits (LiankeBio, China; EK280, EK218, and EK201B) according to the manufacturer’s instructions. Absorbance values were recorded at 450 nm using a microplate reader (Spark10M, TECAN, Switzerland). The levels of the corresponding cytokines were calculated based on the standard curves generated for each assay.

### 2.11 Statistical analysis

Bioinformatic analyses were conducted using OmicStudio platform (https://www.omicstudio.cn/tool) (15). Statistical analyses and graphical representations were performed using GraphPad Prism 8. Data are presented as mean ± standard deviation (SD). Given the small sample size for some analyses, formal normality testing was not performed. Two-sided unpaired Welch’s t-tests were used for comparisons between independent groups without assuming equal variances. Each intervention group was compared with the corresponding irradiation group. P < 0.05 was considered statistically significant.

## 3 Results

### 3.1 UPLC-MS/MS identification of the major components of QHYCF

The chemical profile of the aqueous extract of QHYCF was characterized using UPLC–MS/MS analysis. As illustrated in **Figure 1**, the total ion chromatograms displayed numerous well-separated peaks under both positive and negative ionization modes, reflecting the chemical complexity of the formula and the satisfactory chromatographic resolution achieved in the analysis. Several peaks with relatively strong signal intensities were consistently detected, providing a solid foundation for subsequent compound annotation. Database matching against mzCloud yielded a total of 1101 candidate compounds, of which 593 compounds obtained a comprehensive score above 60. Chromatographic signals with relatively high abundance were further examined by evaluating their peak shapes. After manual inspection and confirmation, 18 representative constituents were ultimately annotated. Detailed information regarding these identified compounds is presented in **Supplementary Material 1**.

**Figure 1:**
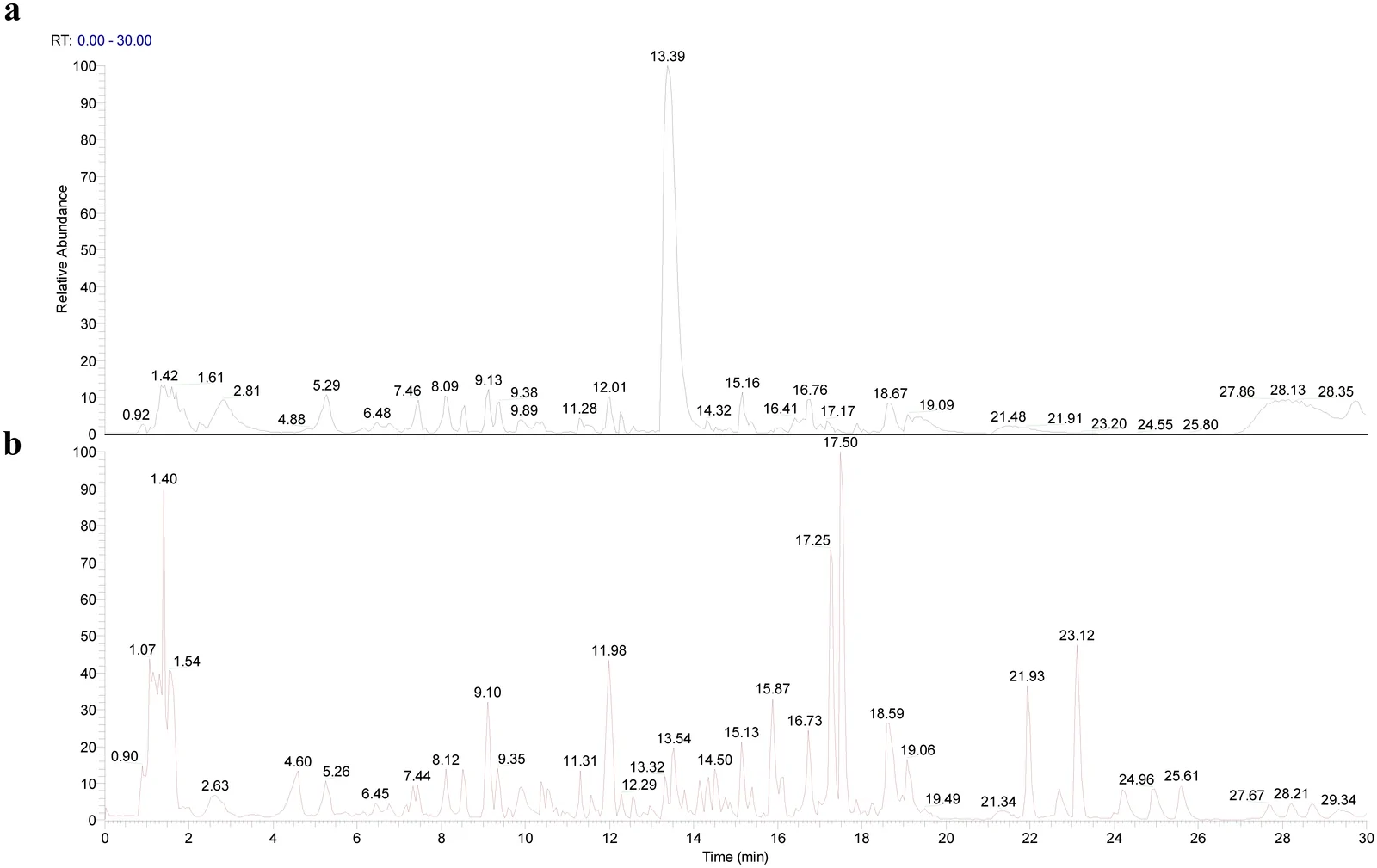
UPLC-MS/MS total ion current chromatograms of QHYCF in positive and negative ion modes **(a)** negative ion mode; **(b)** positive ion mode. Numbers above the peaks indicate retention time (min). Representative compounds corresponding to major peaks are summarized in **Supplementary Material 1**.

### 3.2 ARE model construction and intestinal pathology analysis

The experimental design for the first batch of animals is presented in **Figure 2a**. During the acclimation period, all mice demonstrated steady body weight gain and maintained normal physiological status. After irradiation, animals in the NC group remained in good condition, whereas mice in the IR group exhibited pronounced weight reduction within 7 days post-irradiation (**Figure 2b**). In addition, irradiated mice displayed clinical manifestations including rough fur, decreased mobility, and diarrhea. These symptoms showed gradual recovery after day 7.

**Figure 2:**
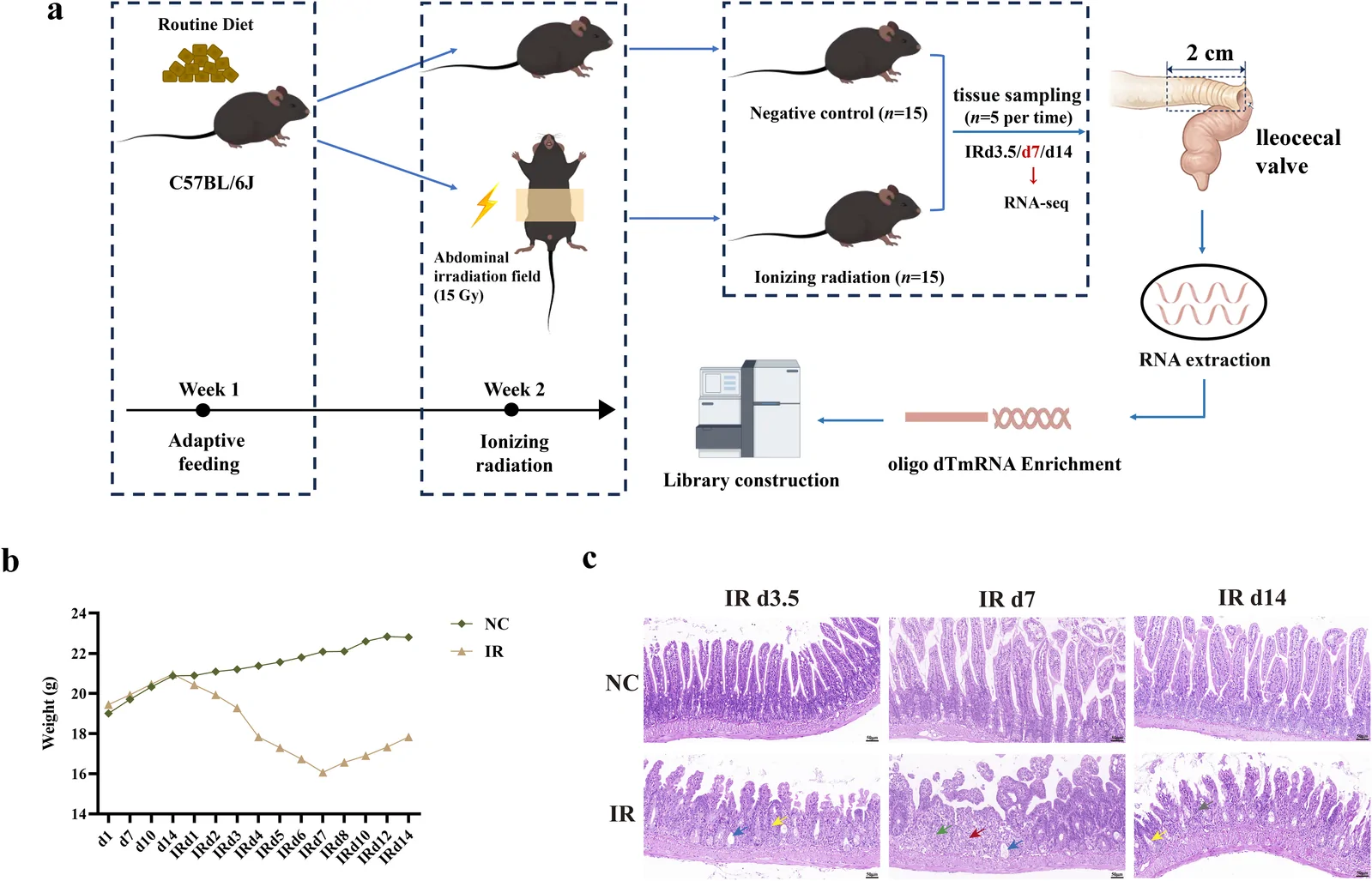
Establishment and validation of the ARE murine model **(a)** the experimental flow for the first batch of animals **(b)** body weight changes of NC vs IR group **(c)** HE staining of intestinal tissue at 3.5, 7, and 14 days after irradiation (x200). Localized abdominal irradiation was performed using a clinically defined radiation field with a linear accelerator, without lead shielding. Arrows: blue = gland dilation, yellow = gland hyperplasia, green = connective tissue replacement, red = lymphocyte infiltration, gray = granulocyte infiltration. Abbreviations: NC, normal control; IR, irradiation.

Histopathological evaluation using H&E staining (**Figure 2c**) showed that intestinal architecture in the NC group remained intact. In contrast, the IR group displayed obvious pathological alterations. Specifically, gland enlargement was detected on day 3.5, while glandular dilation, lymphocyte infiltration, and fibroplasia became apparent on day 7. By day 14, focal inflammatory infiltration and glandular hyperplasia were still observed, indicating sustained intestinal injury. The key pathological features shown in **Figure 2** are marked with color-coded arrows.

### 3.3 Transcriptomic analysis reveals activation of immune and inflammatory pathways following irradiation

Transcriptomic analysis of intestinal tissues collected 7 days after irradiation revealed substantial transcriptional differences between the IR and NC groups. Using DESeq2 analysis (16) with thresholds of |log_2_FC| > 2 and FDR < 0.05, a total of 663 differentially expressed genes (DEGs) were identified, including 378 upregulated and 285 downregulated genes (**Figure 3a**). The DEG heatmap (**Figure 3b**) demonstrated a clear separation between the two groups.

**Figure 3:**
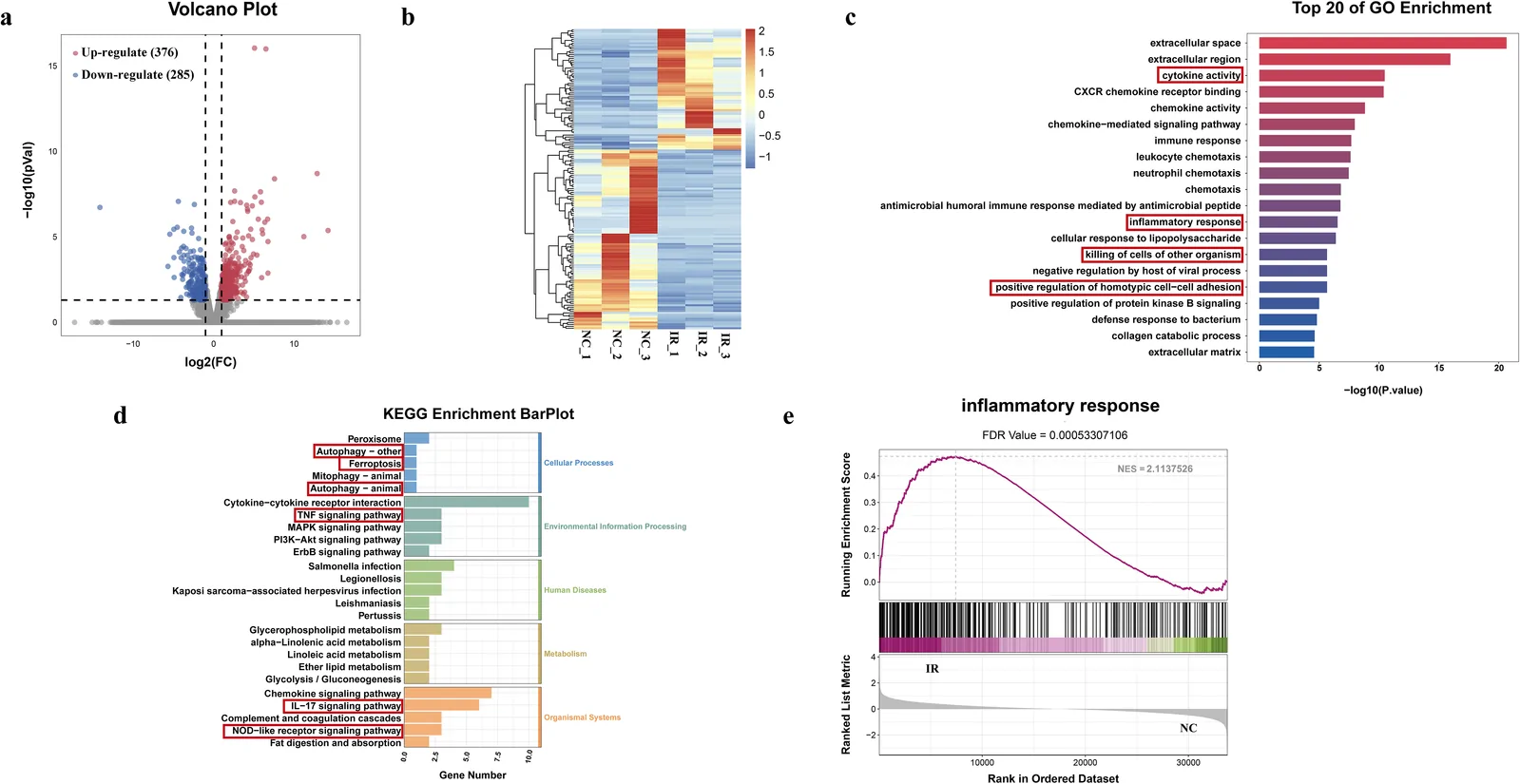
Transcriptomic changes in intestinal tissue induced by radiation (IR vs. NC) **(a)** volcano plot of DEGs (IR vs NC) **(b)** Heatmap of DEGs showing distinct clustering of NC and IR samples based on gene expression patterns **(c)** Top 20 GO enrichment terms of DEGs **(d)** KEGG pathway enrichment of DEGs. **(e)** GSEA plot showing enrichment of inflammatory response gene sets. Abbreviations: DEGs, differentially expressed genes; NES normalized enrichment score; FDR, false discovery rate.

Gene Ontology (GO) enrichment analysis (17) indicated that these DEGs were primarily associated with biological processes related to immune responses, inflammatory regulation, and cytokine-mediated signaling pathways (**Figure 3c**). Several genes were also linked to functions such as homotypic intercellular adhesion and pathogen elimination, suggesting that radiation exposure may compromise intestinal barrier function and innate immune defense mechanisms. KEGG pathway enrichment analysis (18) demonstrated that the DEGs were mainly involved in signaling pathways associated with pro-inflammatory responses, innate immune activation, and cellular stress signaling (**Figure 3d**). Pathways closely related to inflammatory signaling and innate immune recognition were emphasized with red boxes in **Figure 3d**. Consistent with these findings, Gene Set Enrichment Analysis (GSEA) revealed significant enrichment of inflammatory response-related gene sets in the IR group (NES = 2.1137, FDR = 0.0005) (**Figure 3e**). These transcriptomic findings suggest that ARE is closely associated with inflammatory activation and immune-related signaling alterations.

### 3.4 Transcriptomic analysis highlights involvement of the NOD-like receptor signaling pathway in ARE

Transcriptomic analysis indicated prominent alterations in innate immune signaling pathways during ARE. Based on this finding, we focused on the potential involvement of the NOD-like receptor signaling pathway in ARE. GSEA revealed significant enrichment of the NOD-like receptor signaling pathway in the IR group (**Figure 4a**). As a key component associated with this pathway, NLRP3 expression changes were particularly prominent (**Figure 4b**). To validate the transcriptome findings, we assessed the dynamic expression of NLRP3 in intestinal tissues across multiple time points at both the mRNA and protein levels. RT-qPCR analysis (**Figure 4c**) showed that NLRP3 mRNA expression was slightly upregulated on day 3.5 post-irradiation (*P* < 0.01) and day 7 (*P* < 0.01), whereas the difference was no longer statistically significant on day 14 (*P* > 0.05).

**Figure 4:**
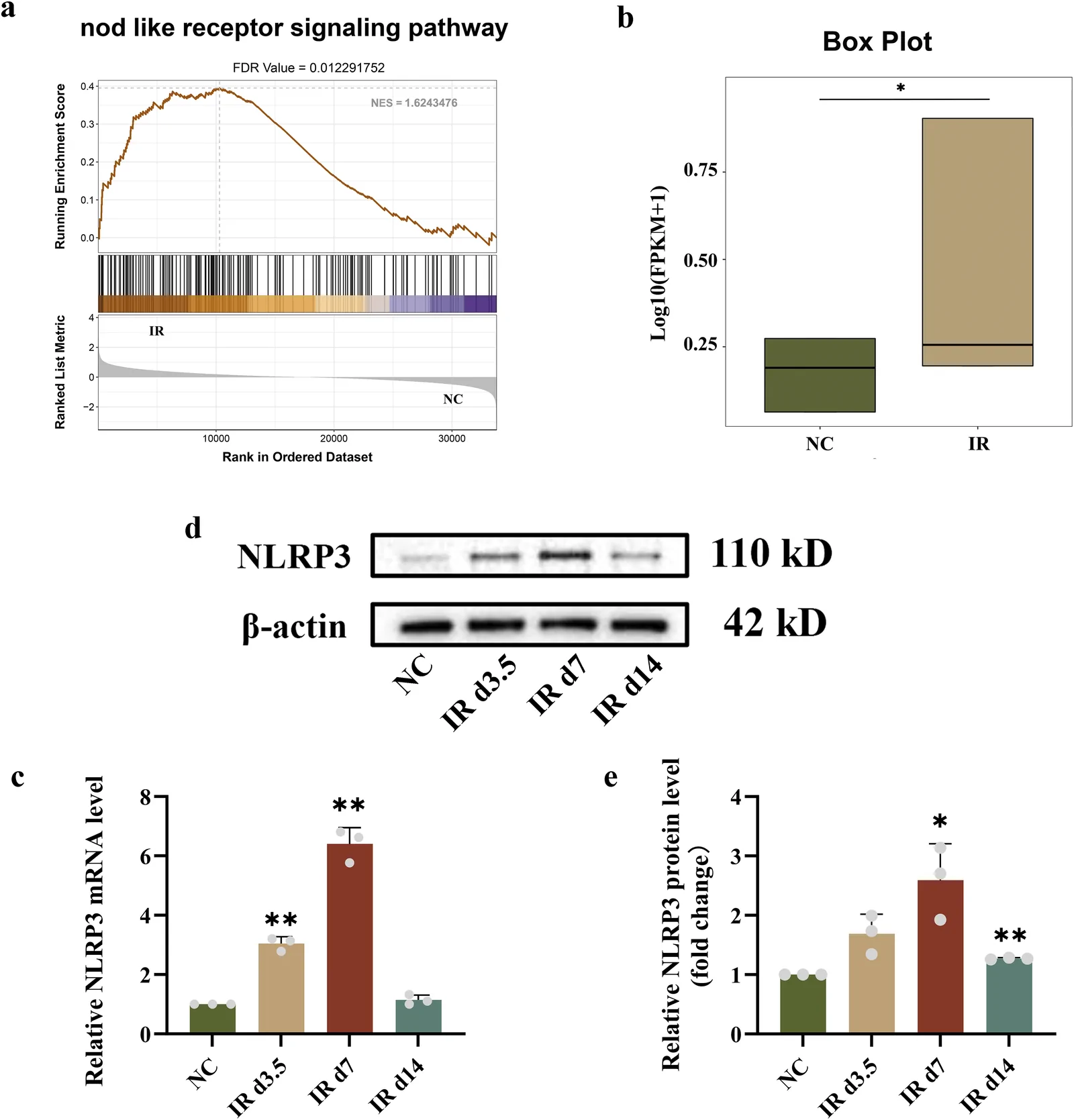
Transcriptomic and molecular characterization of NLRP3 expression in ARE **(a)** GSEA enrichment for NOD-like receptor pathway **(b)** box plot of NLRP3 mRNA expression **(c)** relative mRNA levels of NLRP3 at different time points after irradiation **(d)** representative western blot images of NLRP3 protein expression at different time points after irradiation **(e)** quantification of NLRP3 protein levels from individual mouse samples (*n* = 3 per group, randomly selected from five mice at each time point, mean ± SD). *P < 0.05, **P < 0.01 vs NC (normal control). Protein levels were normalized to β-actin and expressed as fold change relative to NC. Each dot represents one individual mouse.

Western blot analysis (**Figures 4d, e**) showed an increasing trend in NLRP3 protein expression at day 3.5 after irradiation, although the difference did not reach statistical significance. NLRP3 protein expression was significantly elevated at day 7 (*P* < 0.05) and remained significantly higher than that in the NC group at day 14 (*P* < 0.01). Overall, the protein expression profile generally paralleled the dynamic changes observed at the mRNA level. These findings collectively suggest that activation of the NOD-like receptor signaling pathway, accompanied by increased NLRP3 expression, is associated with inflammatory responses during the early stages of ARE.

### 3.5 QHYCF attenuates intestinal pathological injury in ARE mice

After the preliminary characterization of the pathogenic mechanisms of ARE, intervention experiments were performed using different concentrations of QHYCF. The experimental workflow is shown in **Figure 5a**. Histopathological assessment of intestinal tissues was conducted at multiple time points after irradiation, and representative pathological features observed under different treatments are indicated by color-coded arrows in **Figure 5b**.

**Figure 5:**
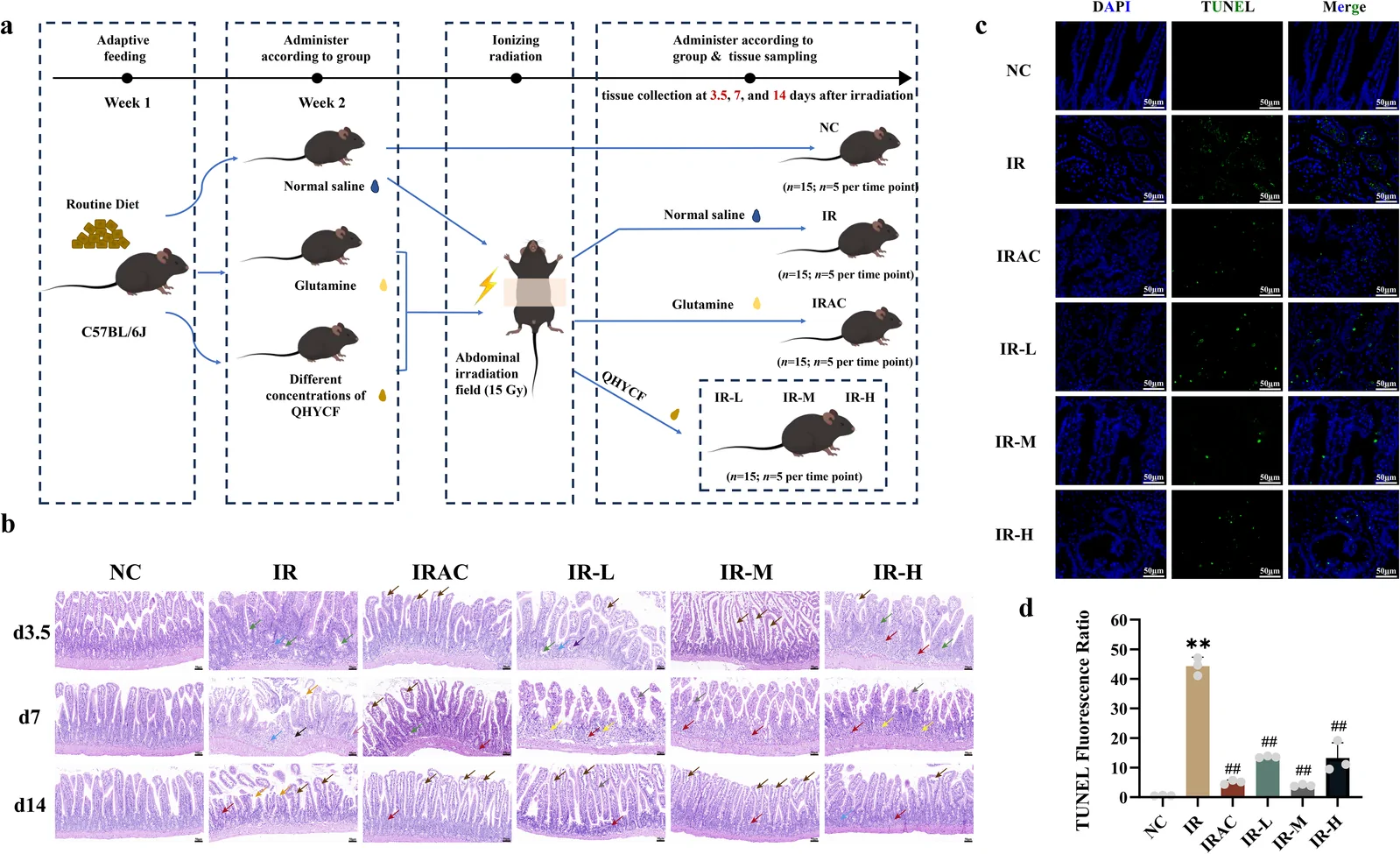
Experimental design and histopathological evaluation of QHYCF intervention in ARE mice **(a)** the experimental flow for the second batch of animals **(b)** HE staining of intestinal tissue at IRd3.5, 7, and 14 (x200). Arrows: Brown = Separation between the intestinal villous epithelium and the lamina propria; Green = Enlarged intestinal gland volume with mild hyperplasia; Purple = Necrotic cell debris within the glands; Blue = Small focal lymphocyte infiltration; Red = Granulocyte infiltration; Orange = Mucosal epithelial cell necrosis and shedding; Black = Glandular replacement by proliferative repair of connective tissue; Pink = Abnormal gland morphology; Gray = Pale-staining cytoplasm with a loose structure; Yellow = Reduced gland number and loose glandular arrangement **(c)** representative TUNEL staining images of each group (blue, DAPI; green, TUNEL-positive apoptotic cells) **(d)** quantification of the apoptotic index (quantification was performed using three randomly selected mice per group, percentage of TUNEL-positive nuclei over total DAPI-positive nuclei). *P < 0.05, **P < 0.01 vs NC; #*P* < 0.05 vs IR. NC, normal control; IR, irradiation; IRAC, irradiation + glutamine treatment; IR-L, irradiation + low-dose QHYCF; IR-M, irradiation + medium-dose QHYCF; IR-H, irradiation + high-dose QHYCF.

In the NC group, the intestinal architecture remained intact without detectable pathological abnormalities. In contrast, mice in the IR group developed progressive tissue damage. On day 3.5, enlargement of intestinal glands and irregular hyperplasia accompanied by lymphocyte infiltration in the lamina propria were observed, suggesting the initiation of inflammatory injury. By day 7, intestinal pathology became more severe, characterized by a reduction in glandular structures, epithelial cell necrosis, and granulocyte infiltration. Although partial recovery was observed on day 14, structural abnormalities such as epithelial layer separation remained evident, indicating incomplete tissue repair.

In comparison, mice receiving QHYCF treatment exhibited varying degrees of histological improvement across the examined time points. On day 3.5, only mild epithelial detachment was observed in the IRAC and IR-M groups, while the IR-L and IR-H groups showed reduced inflammatory infiltration relative to the untreated IR group. On day 7, the IR-M group displayed nearly normal histological morphology, and all treatment groups demonstrated less severe pathological injury than the IR group. By day 14, clear signs of inflammation resolution and epithelial regeneration were observed in all intervention groups.

Collectively, these findings indicate that QHYCF treatment alleviates radiation-induced intestinal damage, accompanied by reduced inflammatory infiltration and enhanced epithelial repair.

### 3.6 QHYCF reduces radiation-induced epithelial apoptosis in intestinal tissue

To further confirm intestinal injury beyond histological H&E staining, TUNEL staining was performed to detect epithelial cell apoptosis at day 7 post-irradiation. As shown in **Figure 5c**, almost no TUNEL-positive cells were observed in the NC group, whereas the IR group exhibited a marked increase in apoptotic nuclei. Both glutamine (IRAC) and QHYCF treatment reduced apoptosis to varying degrees, with the medium dose of QHYCF showing the most pronounced effect. Quantitative analysis of the apoptotic index (TUNEL^+^/DAPI^+^) revealed a significant elevation in the IR group compared with NC, which was significantly attenuated by glutamine and all doses of QHYCF treatment (**Figure 5d**). In summary, QHYCF-treated groups showed reduced epithelial apoptosis, consistent with the milder histopathological injury observed in other analyses.

### 3.7 QHYCF attenuates ARE accompanied by reduction of NLRP3 expression and preservation of Claudin-1

Western blot analysis revealed that radiation exposure markedly increased NLRP3 (**Figures 6a, c–e**) expression while suppressing the expression of Claudin-1 (**Figures 6b, f–h**). On IR d3.5, NLRP3 protein expression was significantly upregulated in the IR group (*P* < 0.05), whereas Claudin-1 levels were significantly downregulated (*P* < 0.05). Treatment with QHYCF effectively counteracted these alterations: medium-dose QHYCF (IR-M) significantly downregulated NLRP3 expression (*P* < 0.05) and upregulated Claudin-1 (*P* < 0.05) at the same time point. By day 7, NLRP3 expression remained elevated in the IR group (*P* < 0.05), accompanied by a further reduction in Claudin-1 expression (*P* < 0.01). At this stage, both medium- and high-dose QHYCF treatments significantly inhibited NLRP3 expression (*P* < 0.05), although their effect on Claudin-1 expression was limited. On day 14, NLRP3 and Claudin-1 expression levels remained abnormal in the IR group (*P* < 0.05 for both); however, the IRAC, IR-M, and IR-H groups demonstrated significant restoration of Claudin-1 expression (*P* < 0.05). These results suggest that QHYCF treatment is associated with reduced NLRP3 expression, restoration of Claudin-1 expression, and attenuation of radiation-induced intestinal injury.

**Figure 6:**
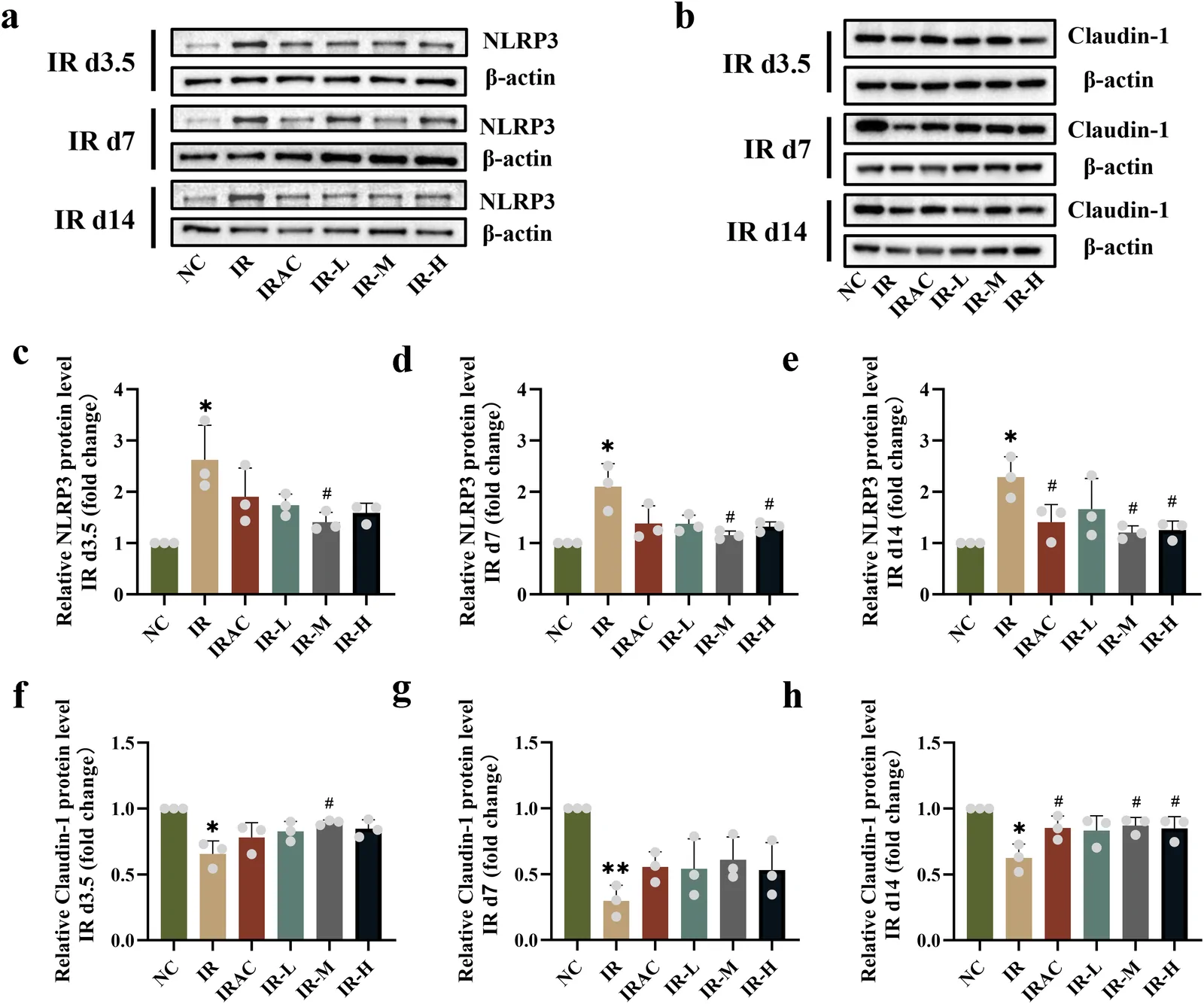
QHYCF is associated with reduced NLRP3 expression and preservation of Claudin-1 **(a–b)** representative Western blot images of NLRP3 **(a)** and Claudin-1 **(b)** protein expression in intestinal tissues at different time points after irradiation **(c–e)** quantitative analysis of NLRP3 protein expression from individual mouse samples (*n* = 3 per group, randomly selected from five mice at each time point, mean ± SD) **(f–h)** quantitative analysis of Claudin-1 protein expression from individual mouse samples (*n* = 3 per group, randomly selected from five mice at each time point, mean ± SD) Protein levels were normalized to β-actin and expressed as fold change relative to NC *P < 0.05, **P < 0.01 vs NC; #*P* < 0.05 vs IR. Protein levels were normalized to β-actin from the corresponding time point and expressed as fold change relative to NC. Each dot represents one individual mouse.

### 3.8 QHYCF attenuates radiation-induced pro-inflammatory cytokine responses

ELISA analysis demonstrated that radiation significantly increased serum levels of pro-inflammatory cytokines (**Figure 7**). On day 3.5 after irradiation, the levels of IFN-γ, IL-18, and IL-1β were significantly elevated in the IR group compared with the NC group (*P* < 0.01). QHYCF treatment partially reduced these cytokine elevations, with significant effects observed in specific dose groups. On day 7, serum levels of IFN-γ, IL-18, and IL-1β remained significantly increased in the IR group (*P* < 0.01), whereas QHYCF treatment attenuated these inflammatory responses across different cytokines. By day 14, inflammatory cytokine levels in the IR group remained elevated, whereas QHYCF treatment showed a tendency to decrease these cytokine levels. These findings suggest that QHYCF treatment was associated with sustained attenuation of radiation-induced inflammatory responses during ARE progression.

**Figure 7:**
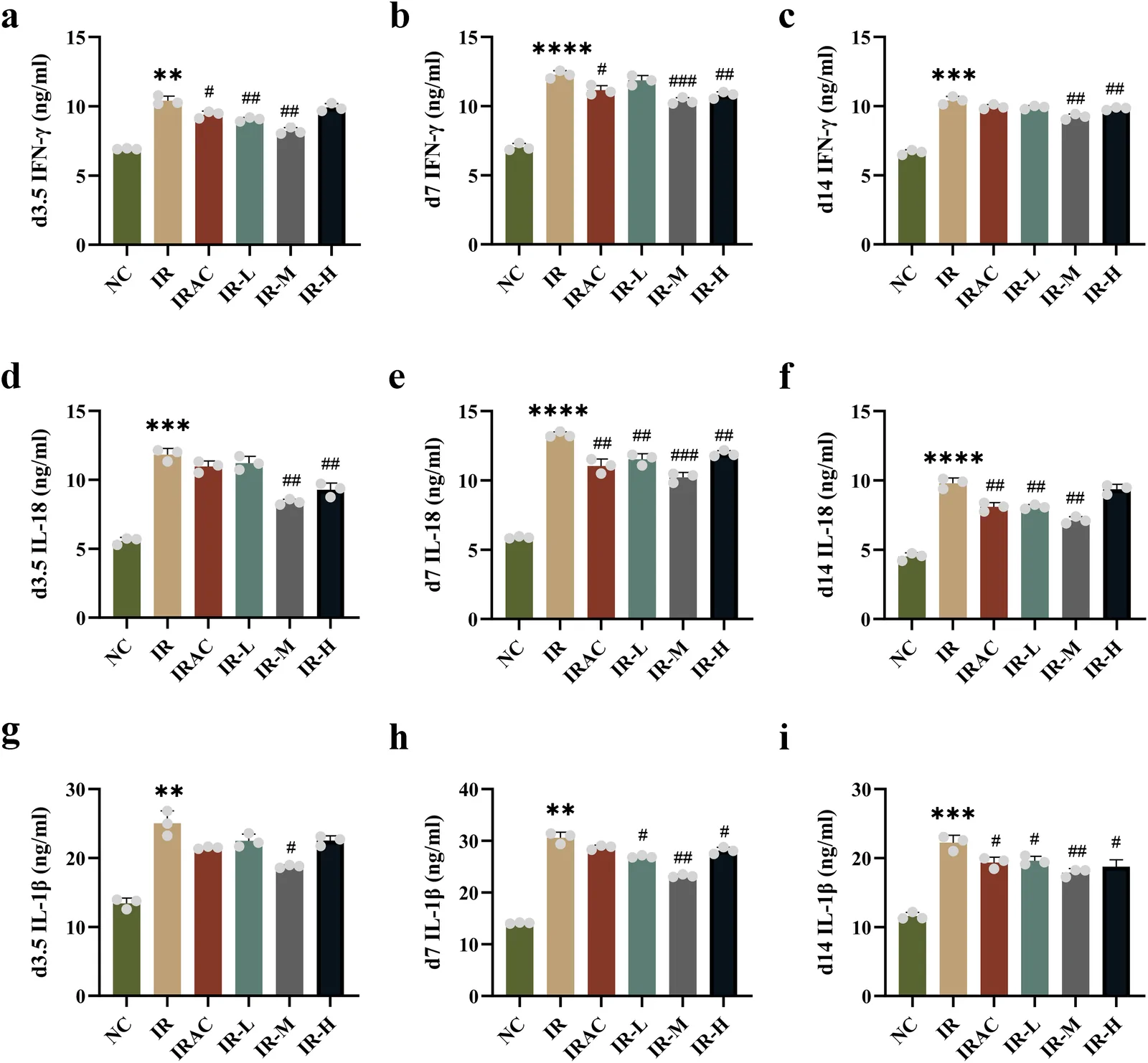
QHYCF reduces serum cytokines in ARE mice **(a–c)** Serum levels of IFN-γ **(d–f)** Serum levels of IL-18 **(g-i)** Serum levels of IL-1β at IR d3.5, IR d 7, IR d 14 (*n* = 3 per group, randomly selected from five mice at each time point, mean ± SD) **P < 0.01, ***P < 0.001, ****P < 0.0001 vs NC; ^#^*P* < 0.05, ^##^*P* < 0.01, ^###^*P* < 0.001 vs IR. Each dot represents one individual mouse.

## 4 Discussion

Radiotherapy remains a cornerstone of cancer treatment, with more than 50% of patients undergoing it during the course of their illness (19). Despite advancements in radiotherapy precision and technology, collateral damage to surrounding healthy tissues is still inevitable (1). Among these, radiation-sensitive tissues such as the small intestine and bone marrow are particularly vulnerable to radiation-induced injury (20). ARE is a common and significant complication of abdominal or pelvic radiotherapy. It results in epithelial damage, gastrointestinal dysfunction, and clinical manifestations including diarrhea, gastrointestinal bleeding, and secondary infections, all of which can severely impair patients’ quality of life (21).

Transcriptomic profiling of intestinal tissues following irradiation provided insight into the molecular alterations underlying radiation-induced intestinal injury by comparing the NC and IR groups. Pathway-level analyses revealed significant enrichment of immune- and inflammation-related pathways, with the NOD-like receptor signaling pathway emerging as a prominent feature. Although inflammasomes such as NLRP1, AIM2, and NLRC4 have been implicated in intestinal inflammation (22), their specific roles in radiation-induced enteritis remain unclear. In contrast, accumulating evidence suggests that NLRP3 inflammasome activation plays an important role in radiation-induced intestinal injury (23). Integrating transcriptomic profiling with molecular validation in a well-established C57BL/6 mouse model, we consistently observed increased NLRP3 expression and activation of inflammation-related signaling pathways. While previous studies have implicated NLRP3 inflammasome activation in radiation-induced tissue injury (23, 24), the temporal dynamics of NLRP3 expression *in vivo* remain poorly characterized. Our previous study (25) provided pharmacological evidence supporting the involvement of NLRP3 inflammasome signaling in radiation-induced intestinal injury using the selective NLRP3 inhibitor MCC950. In the present study, we further demonstrated that NLRP3 expression remained elevated during the peak phase of intestinal injury, accompanied by pronounced inflammatory responses and histopathological damage. Together with our previous pharmacological observations, these findings suggest that NLRP3-associated inflammatory signaling may participate in the inflammatory alterations observed during ARE progression. Nevertheless, the transcriptomic data primarily represent ARE-associated molecular signatures rather than direct evidence of QHYCF-mediated transcriptomic regulation. Furthermore, this study was not designed to determine whether NLRP3 is required for the protective effects of QHYCF. Therefore, the observed relationships should be interpreted as associative rather than causal. In addition, the small number of biological replicates used for some molecular assays limits the robustness of statistical inference, and these findings require confirmation in larger independent experiments.

As a classical TCM formulation, QHYCF was associated with improvements in histological injury, inflammatory responses, and epithelial barrier integrity in ARE. Specifically, QHYCF treatment was accompanied by reduced expression of NLRP3, decreased levels of pro-inflammatory cytokines (IL-1β, IL-18, and IFN-γ), and partial restoration of Claudin-1 expression in irradiated intestinal tissue. Moreover, radiation enteritis is closely associated with apoptosis (26). TUNEL staining further confirmed a substantial elevation of epithelial apoptosis in the irradiated group, whereas QHYCF treatment was associated with a marked reduction in apoptosis, particularly at the medium dose. These findings suggest that the attenuation of intestinal injury may involve concurrent modulation of inflammatory activity, epithelial cell survival, and barrier-related molecular features. While several TCM formulations have shown promise in managing inflammatory bowel conditions (27), the present study extends these observations to radiation-induced enteropathy by integrating transcriptomic and histopathological evidence. The magnitude of improvement varied among different doses, with the medium-dose group showing relatively pronounced effects in several pathological and molecular assessments. These observations are consistent with the non-linear dose-response characteristics commonly reported for multi-component herbal formulations, emphasizing the need for future studies to identify key bioactive components and optimize formulation ratios. Nevertheless, this study has an important limitation in that a QHYCF-only control group was not included. Because the baseline effects of QHYCF on healthy, non-irradiated animals were not evaluated, it cannot be fully excluded that some observed molecular and histopathological changes may reflect the baseline effects of QHYCF under normal physiological conditions. Therefore, the protective effects of QHYCF observed in this study should be interpreted within the context of radiation-induced intestinal injury.

NLRP3 inflammasome activation is known to be triggered by DAMPs, mitochondrial ROS, potassium efflux, and NF-κB signaling (28). Once activated, NLRP3 mediates the maturation and secretion of pro-inflammatory cytokines such as IL-1β and IL-18, together with the initiation of downstream inflammatory cascades. While transient activation may serve a protective role, persistent or excessive activation of the NLRP3 inflammasome has been implicated in disrupting intestinal homeostasis and impairing epithelial barrier integrity (29, 30). The epithelial cell monolayer and its tight junctions, which rely on Claudin family proteins for barrier integrity, serve as the frontline mechanical defense against luminal pathogens and inflammatory insults (31). In this study, QHYCF treatment was associated with reduced inflammatory responses and preservation of Claudin-1 expression, suggesting an association between attenuation of inflammatory responses and preservation of epithelial barrier integrity. Nevertheless, the present study was not designed to establish a causal relationship between NLRP3 activation and epithelial barrier dysfunction. Although we evaluated NLRP3 expression together with circulating inflammatory cytokines (serum IL-1β and IL-18 levels), classical inflammasome activation markers such as cleaved caspase-1, ASC speck formation, and GSDMD-N were not assessed in this study. Future studies incorporating these markers and genetic or pharmacological intervention approaches are warranted to further clarify the role of NLRP3 signaling.

Disruption of these junctions leads to increased intestinal permeability, facilitating inflammatory responses and exacerbating tissue damage (32). Although the upstream regulatory mechanisms responsible for the observed changes in NLRP3 expression were not investigated in the present study, whether QHYCF directly regulates these signaling pathways remains to be determined. The temporal expression patterns of NLRP3 and Claudin-1 reflect dynamic inflammatory and epithelial barrier alterations during ARE progression. Additionally, restoration of Claudin-1 expression suggests that the protective effects of QHYCF may not be limited to attenuation of inflammation-associated alterations. Previous studies suggested that Wnt/β-catenin (33) or MAPK pathways may play roles in epithelial proliferation and tight junction integrity, which warrants further investigation. Collectively, these findings support an association between NLRP3-associated inflammatory alterations, epithelial barrier dysfunction, and the progression of ARE. Further studies using clinical samples are required to determine whether these molecular alterations have potential clinical relevance.

## 5 Conclusion

Radiation exposure induces robust intestinal inflammation and disrupts epithelial barrier integrity. In this study, QHYCF administration was associated with attenuation of radiation-induced intestinal injury, accompanied by reduced expression of NLRP3, decreased levels of pro-inflammatory cytokines, and increased Claudin-1 expression. These findings suggest that inflammatory regulation and epithelial barrier alterations are closely involved in the progression of ARE. Notably, these molecular alterations provide additional insights into the inflammatory and epithelial barrier changes occurring during ARE progression. However, further mechanistic and clinical studies are required to clarify causal mechanisms and evaluate the translational relevance of these findings.

## Data Availability

The datasets presented in this study can be found in online repositories. The names of the repository/repositories and accession number(s) can be found below: PRJNA1245597 (SRA).
