# Supplementary material for: Transcriptomic profiling identifies NLRP3-associated inflammatory signatures in acute radiation enteritis and characterizes the protective effects of Qing Hua Yi Chang formula
Source: Front Immunol. 2026 Sep 16;17:1890536. doi: 10.3389/fimmu.2026.1890536 (PMC13623686; doi:10.3389/fimmu.2026.1890536)
Supplement: Supplementary file 1 [file Table1.docx]

Supplementary Table 1 The primer sequences of NLRP3

| Primer | Primer sequence (5’-3’) | Species |
| --- | --- | --- |
| NLRP3 | F: CCAGACCTCCAAGACCACTACG | Mus musculus |
|  | R: CAGAGAAGAGATGCTCCTCAATGC |  |
| β-Actin | F: ACTGCCGCATCCTCTTCCTC | Mus musculus |
|  | R: AACCGCTCGTTGCCAATAGTG |  |
