# Supplementary material for: Transcriptomic profiling identifies NLRP3-associated inflammatory signatures in acute radiation enteritis and characterizes the protective effects of Qing Hua Yi Chang formula
Source: Front Immunol. 2026 Sep 16;17:1890536. doi: 10.3389/fimmu.2026.1890536 (PMC13623686; doi:10.3389/fimmu.2026.1890536)
Supplement: Supplementary file 2 [file Table2.docx]

**1. Methods**

***Identification of Active Compounds in QHYCF***

The chemical constituents of QHYCF were analyzed by ultra-performance liquid chromatography coupled with tandem mass spectrometry (UPLC-MS/MS) under the following chromatographic and mass spectrometric conditions.

Chromatographic separation was performed using an Accucore HPLC AQ-C18 column (150 × 2.1 mm, 1.8 μm) maintained at 35 °C. The mobile phase consisted of 0.1% formic acid in water (A) and methanol (B), with a flow rate of 0.30 mL/min.

Mass spectrometric analysis was carried out on a Q Exactive Orbitrap high-resolution mass spectrometer (Thermo Fisher Scientific, USA) equipped with an electrospray ionization (ESI) source. The MS parameters were as follows: spray voltage, 3.2 kV; capillary temperature, 300 °C; sheath gas (nitrogen), 40 Arb; auxiliary gas (nitrogen), 15 Arb at 350 °C; collision gas, high-purity argon (≥99.999%); and normalized collision energy (NCE) values of 30, 40, and 60. The scan range was set to m/z 100.0–1500.0, and the data acquisition time was 30.0 minutes.

The obtained data were processed using Compound Discoverer 3.3 (Thermo Fisher Scientific), and compounds were identified based on spectral matching against the mzCloud database, with a match score cutoff set at >60.

**2.Results**

A total of 1101 compounds were matched against the mzCloud database, among which 593 compounds had match scores greater than 60. The peak shapes of high-abundance chromatographic signals were manually inspected, and corresponding peaks were labeled. As a result, eighteen representative compounds were successfully identified. Supplementary Table 2 listed the details of these compounds.

| No. | the identified compounds | Formula | Adduct ion | *t*_R_/min | *m/z* |
| --- | --- | --- | --- | --- | --- |
| 1 | D-(+)-Pipecolinic acid | C6H11NO2 | [M+H]+ | 1.61 | 130.08641 |
| 2 | Naringeninchalcone | C15H12O5 | [M+H] + | 12.01 | 273.07568 |
| 3 | Oryzalin | C12H18N4O6S | [M-H]- | 4.60 | 345.08255 |
| 4 | Citric acid | C6H8O7 | [M-H]- | 8.12 | 191.01909 |
| 5 | 2,5-bis(4-hydroxy-3-methoxyphenyl)-3,4-  dimethyloxolan-3-ol | C20H24O6 | [M+H+MeO]+ | 11.31 | 383.14859 |
| 6 | 2-[4-(tert-butyl)phenyl]-5-methylperhydroisoindole-  1,3-dione | C19H25NO2 | [M+H]+ | 11.98 | 300.1956 |
| 7 | Lariciresinol 4-O-glucoside | C26H34O11 | [M-H]- | 11.28 | 521.20319 |
| 8 | 7-hydroxy-2-(4-hydroxyphenyl)-5-methoxy-3,4-  dihydro-2H-1-benzopyran-4-one | C16H14O5 | [2M+FA-H]- | 13.39 | 433.11368 |
| 9 | 2,3-dihydroxypropyl 12-methyltridecanoate | C17H34O4 | [M+Na]+ | 19.09 | 325.23489 |
| 10 | L-Phenylalanine | C9H11NO2 | [M+NH4]+ | 5.26 | 166.08623 |
| 11 | 4-(2-Hydroxyethyl)-2-methoxyphenyl β-D-  glucopyranoside | C15H22O8 | [M+H]+ | 9.10 | 348.16492 |
| 12 | Coumarin | C9H6O2 | [M+H]+ | 9.35 | 147.04401 |
| 13 | 4-Hydroxybenzaldehyde | C7H6O2 | [M+H]+ | 9.35 | 123.04429 |
| 14 | Phenylacetaldehyde | C8H8O | [M-H]- | 9.35 | 119.04857 |
| 15 | Eucalyptol | C10H18O | [M+H-H2O]+ | 12.29 | 137.13257 |
| 16 | Baicalin | C21H18O11 | [M+H]+ | 15.13 | 447.09213 |
| 17 | 2-(8-Hydroxy-4a,8-dimethyldecahydro-2-  naphthalenyl)acrylic acid | C15H24O3 | [M+Na]+ | 17.50 | 275.16147 |
| 18 | 1-Stearoylglycerol | C21H42O4 | [M+H]+ | 23.12 | 359.31494 |

Supplementary Table 2 Peak assignments of components in the QHYCF extract
